# Sensory sensitivity and its relationship with adult attachment and parenting styles

**DOI:** 10.1371/journal.pone.0209555

**Published:** 2019-01-09

**Authors:** Grace Branjerdporn, Pamela Meredith, Jenny Strong, Mandy Green

**Affiliations:** 1 School of Rehabilitation and Health Sciences, Faculty of Health and Behavioral Sciences, The University of Queensland, Brisbane, Queensland, Australia; 2 School of Health, Medical and Applied Sciences, Central Queensland University, Rockhampton, Queensland, Australia; Technion Israel Institute of Technology, ISRAEL

## Abstract

Parenting styles vary in levels of both warmth and control, with evidence that type of parenting behavior is linked with social-emotional and other developmental outcomes for children. There are well-established associations between adult attachment and parenting styles. Given emerging evidence that people with different attachment patterns vary in how they receive and modulate sensory information, there are potential implications for parenting which have rarely received research attention. This cross-sectional study investigates the links between parenting style and parental sensory sensitivity, and the possible mediating role of parental sensory sensitivity in the relationship between adult attachment and parenting styles. A convenience sample of 155 parents of children aged 4–12 years old completed an online survey measuring: adult attachment (Experiences in Close Relationships-Modified 16-item Scale), sensory sensitivity (Highly Sensitive Persons Scale-Shortened Version), and parenting styles (Parenting Styles and Dimensions Questionnaire). Correlation, regression and mediation analyses were conducted. Analyses revealed that parents who reported more attachment insecurity also reported higher levels of parental sensory sensitivity, and more authoritarian and/or permissive (non-optimal) parenting styles. Parental sensory sensitivity was found to fully mediate the relationship between attachment avoidance and permissive parenting, and to partially mediate the relationship between attachment anxiety and both authoritarian and permissive parenting. This study represents the first quantitative evidence for associations between parental sensory sensitivity and parenting styles, and the mediating effect of parental sensory sensitivity on the known relationship between attachment insecurity and parenting. Awareness of a parent’s level of sensory sensitivity, in addition to his/her attachment style, may assist in developing effective strategies to meet both the parent’s and child’s needs and support the parent-child relationship.

## Introduction

Increasing evidence linking warm, responsive and democratic parenting behaviors to positive behavioral and developmental outcomes for primary-school aged children has highlighted the need for improved understanding of parental factors that influence effective parenting behaviors [1–3]. It is well established that adult attachment patterns are key determinants of parenting behaviors (for a review, see [4]), and emerging evidence reveals that different attachment patterns vary in how they receive and modulate sensory information [5–8]. Nevertheless, little is known about the influence of a parent’s sensory-processing patterns (i.e., the manner in which a person receives, modulates and responds to sensory information) [9, 10] on parenting. Atypical sensory-processing patterns have been linked with a range of suboptimal factors in adults, including fewer perceived social supports, reduced health-related quality of life [11], and anxiety [6, 12]. While theoretical relationships have been proposed between parental sensory-processing and parenting style [13, 14], only one study [15] (discussed below) has been undertaken to date. With recent increases in evidence for sensory-informed interventions [13, 14], gaining an improved understanding of these relationships may highlight interventions to promote more effective coping strategies and parenting behaviors, potentially leading to more positive outcomes for children in the short and longer term. Each of these factors will be discussed below.

### Sensory sensitivity

People respond to sensory information in different ways, with some people more or less sensitive (over/under-responsive) to sensory stimuli than others [16]. Two theoretical models developed to measure a person’s sensory processing sensitivity are Dunn’s [17] Model of Sensory Processing, and Aron and Aron’s [9] Highly Sensitive Person (HSP) construct. Both models consider sensory processing sensitivity to be a trait, an aspect of temperament, and are discussed below.

According to Dunn’s [17] Model of Sensory Processing, a person’s sensory sensitivity is based on a combination of his/her neurological threshold and behavioral response. The threshold is conceptualized as a continuum whereby low thresholds require less stimulation to become alerted while high thresholds require more stimulation [17]. The behavioral response is the second continuum, ranging from active to passive. Based on a person’s neurological threshold and behavioral response, Dunn’s model identifies four sensory processing styles: sensory sensitivity, sensory avoidance, sensory seeking, and low registration [17]. *Sensory seeking* and *low registration* reflect high neurological thresholds, with sensory seekers actively seeking stimulation, and people with low registration adopting passive responses [16]. Se*nsory sensitivity* and *avoidance* reflect low neurological thresholds, with people who are sensory sensitive adopting passive responses, doing little to reduce sensory input, and people who are sensory avoidant employing active responses to limit sensory input [16].

Paralleling Dunn’s low neurological threshold sensory patterns, Aron and Aron’s Highly Sensitive Person (HSP) [5] construct relates to a person’s level of emotional and sensory sensitivity. According to Aron and Aron [9], HSP sensitivity is an innate trait associated with a tendency to more strongly and deeply experience a variety of both internal and external stimuli, “…including sensitivity to subtleties, the arts, caffeine, hunger, pain, change, overstimulation, strong sensory input, others’ moods, violence in the media, and being observed” (p. 361). While moderately high levels of HSP sensitivity may be adaptive (e.g., having greater awareness of other people’s emotions), people with high levels of HSP can become easily distressed by stimulation such as loud noises, intense moods, bright lighting, and busy or chaotic environments [9]. Consequently, highly sensitive individuals may organize their environments and routines to avoid or minimize stimulation [10]. In contrast, people with low levels of HSP sensitivity are generally less aware of their environment and are less likely to become distressed by stimuli, and may organize their environments and routines to seek stimulation [10].

While these two sensory processing models are somewhat distinct, significant positive correlations have been found between Dunn’s sensory sensitivity and sensory avoidance patterns, and Aron and Aron’s measure of sensory sensitivity in a recent study examining the relationships between sensory processing, adult attachment and distress in a healthy adult population [7]. While such findings suggest that Aron and Aron’s HSP sensory sensitivity construct is similar to Dunn’s patterns of sensory avoidance and sensory sensitivity, Aron and Aron’s construct does not specify the form of coping strategy (active/passive) adopted to respond to a low neurological threshold [7].

### Parenting styles

While there are various methods for conceptualizing parenting behaviors, one of the most commonly studied models was proposed by Baumrind [18]. The three parenting styles (authoritative, authoritarian, and permissive), included in Baumrind’s model, vary along the dimensions of warmth and control [18]. *Authoritative* parents demonstrate warmth and responsiveness, as well as a respectful and appropriate level of control. *Authoritarian* parents, in contrast, are highly controlling, and exhibit low levels of warmth. *Permissive* parents display high levels of warmth and little control [18]. Of the three styles, authoritative parenting is suggested to be optimal for developmental outcomes for children in Western cultures [1, 19], whereas authoritarian and permissive parenting styles have been linked to poorer outcomes for children and adolescents, including unfavorable dietary habits [19, 20], substance use [19, 21], intentional or unintentional injury, risky sexual behaviors, and physical inactivity [19].

### Adult attachment

According to attachment theory, early relationships and experiences between a child and his/her primary caregivers are considered to lead to the establishment of internal working models (i.e., emotional, cognitive and behavioral schemas of oneself and ones’ attachment figures) [22]. For example, a child who receives inconsistent or unresponsive care may not perceive himself or herself as worthy of love and attention, or the attachment figure as someone who is warm, caring or reliable. These internal working models, developed in infancy, manifest into either a more secure or more insecure attachment pattern, and can remain largely stable over the lifespan [23, 24]. These attachment patterns may also serve as templates guiding future relationships, including future parental caregiving [22, 25–27].

In adulthood, *securely attached* individuals are confident in their self-worth and the reliability of others, tend to adjust to stressful situations, and have more favorable health outcomes [28]. Attachment insecurity in adulthood is often considered as a composite of the two dimensions of attachment anxiety and attachment avoidance [28]. *Anxiously attached* individuals tend to fear rejection or abandonment, have an excessive need for approval, worry about the availability and closeness of others, and actively engage in strategies to seek proximity, closeness and support [26, 29]. These behaviors are understood as a response to their early experiences of attachment figures who were inconsistently responsive [30]. *Avoidantly attached* individuals may fear dependency and avoid close relationships [27], preferring to be self-reliant as a response to their early experiences of attachment figures who were unresponsive and intolerant of vulnerability [30]. Such individuals actively engage in strategies to avoid proximity and closeness [26]. Attachment patterns may also be conceptualized categorically, with four attachment categories identified: secure, fearful, preoccupied and dismissing [27]. While both approaches are conceptually similar and defensible, it has been argued that dimensional assessment methods offer more accurate representations of the adult attachment construct compared to the categorical approach [31].

### Parenting styles and adult attachment

As proposed by Bowlby [22], a parent’s attachment system is thought to influence parenting behaviors and the quality of care a parent provides. For instance, a parent’s attachment-related behaviors may affect his or her ability to respond appropriately to the needs of his or her child [4]. A study by Millings et al. [32] with a sample of 125 couples with children aged 7–8 years, measured romantic attachment using the 36-item Experiences in Close Relationships [33] and found that attachment anxiety and avoidance were positively associated with both authoritarian and permissive parenting styles. In the second study, by Doinita and Dorina [34] with a sample of 74 Romanian parents with children aged 4–8 years, fearful adult attachment (reflecting high levels of both anxious and avoidant attachment), measured using the Adult Attachment Questionnaire [27], was associated with the permissive parenting style. In a review of the attachment/parenting literature *not* specifically focussing on Baumrinds typology, Jones et al. [4] concluded that evidence supports strong associations between insecure attachment patterns and more negative parenting behaviors (i.e., less warmth, supportiveness and responsiveness).

### Parental sensory sensitivity and parenting style

It is feasible that being highly sensitive to sensory stimuli holds parenting implications. Although sensory sensitivity may be an important component of parenting sensitivity in terms of noticing and accurately identifying children’s behavioral cues and empathizing with emotions, parenting is often a highly stimulating and demanding experience which may be particularly challenging for people with high sensory sensitivity. For example, many developmentally appropriate activities undertaken by children (e.g., imaginative play with elaborate props and plots, messy play with different textured items) may be highly stimulating and unpredictable from a sensory perspective. In addition, when a child is having a tantrum, the parent will receive *auditory* stimuli (e.g., child shouting in a high-pitched loud voice, child crying uncontrollably) and *visual* stimuli (e.g., child unpredictably waving their arms around, child throwing objects), as well as possible *proprioceptive* stimuli (e.g., child pushing the parent, parent picking child up) and *vestibular* stimuli (e.g., parent turning to see if others are looking, parent moving towards the child).

In qualitative research by Turner, Cohn and Koomar [15], mothers who were sensory sensitive reported feeling overwhelmed by their sensory needs so adopted coping behaviors such as managing the environment by restricting play spaces (akin to authoritarian parenting behavior), as well as spending time alone, withdrawing from the child (akin to permissive parenting behavior). When examining the literature more broadly among the adult population, empirical evidence supports links between sensory sensitivity and poorer outcomes on a range of psychosocial factors which could conceivably influence parenting behaviors, including less adaptive responses to others’ moods [35], higher levels of psychological distress [36, 37], use of suboptimal coping strategies [38, 39], and poorer quality of social relationships [11].

### Adult attachment and parental sensory sensitivity

It is theoretically plausible that adult attachment pattern may be related to general sensory sensitivity. For example, consider attachment-related alertness to social cues during interpersonal interactions: *Anxiously* attached adults fear abandonment so tend to be hypervigilant to cues regarding the availability of others [7]. In contrast, *avoidantly* attached individuals attempt to maintain emotional distance so may be particularly sensitive to cues regarding the proximity of others [5]. Consistent with these expectations, recent studies have found consistent associations between sensory sensitivity and attachment insecurity [5–8], particularly attachment anxiety [40].

Although no research has specifically investigated links between *parental* sensory sensitivity and attachment patterns, it is plausible that such links may exist [6]. Parents who are highly sensory sensitive may experience exaggerated responses of the kind typical of their attachment pattern. For example, anxiously attached and sensory sensitive parents may be especially distressed by sensory indicators of their child’s autonomy yet overwhelmed by their child’s physical proximity, resulting in ambivalent parenting behaviors. Sensory sensitive parents with avoidant patterns may be particularly alert to sensory cues of their child’s proximity, resulting in avoiding or distancing behavior. Thus, parental alertness and responses to social cues from, and proximity to, their child may be related to both their attachment pattern and their general sensitivity to stimuli (i.e., sensory sensitivity).

### Parental sensory sensitivity, adult attachment, and parenting style

As noted previously, parents process sensory stimuli during all parent-child interactions, and the capacity to manage and respond adaptively in this sensory-rich environment is a function of both level of sensory sensitivity and attachment security. Consequently, parents who are insecurely attached, when confronted with child-related sensory stimuli, may respond to their child (i.e., parenting style) with more rigid responses (i.e., authoritarian parenting) or withdrawal (i.e., passive parenting). This would plausibly be exacerbated as their levels of either attachment insecurity or sensory sensitivity increase. No studies have yet investigated all three variables together.

In summary, (1) extensive literature indicates links between parents’ attachment pattern and their parenting style [4], (2) emerging evidence suggests links between attachment insecurity and sensory sensitivity [5–8], (3) limited research highlights links between sensory sensitivity and parenting styles [15], and (4) no research has considered all three variables together. There is a need to consider both attachment and sensory variables together in relation to parenting styles. While attachment and sensory variables interact in complex ways moment-to-moment [41], for the purposes of this study, it is argued that sensory sensitivity may mediate the more well established relationship between adult attachment and parenting.

### Aims and hypotheses

The aim of this research is to investigate the associations between insecure adult attachment patterns, Baumrind’s parenting styles, and parental sensory sensitivity [18]. Further, this study provides the opportunity to evaluate the potential mediating role of parental sensory sensitivity in the relationship between attachment insecurity and parenting styles. To our knowledge, this is the first quantitative study to consider parental sensory sensitivity when investigating parenting styles, and the first study to consider both sensory sensitivity and attachment in relation to parenting style.

The following hypotheses are proposed:

1. Higher levels of adult attachment avoidance and adult attachment anxiety will be associated with higher levels of parental sensory sensitivity.
2. Higher levels of adult attachment avoidance and adult attachment anxiety will be associated with more authoritarian and permissive parenting styles.
3. Higher levels of parental sensory sensitivity will be associated with more authoritarian or permissive parenting styles.
4. Identified relationships between adult attachment avoidance/anxiety and parenting style will be mediated by parental sensory sensitivity.

## Methods

### Participants and data collection

Ethical approval was obtained from the School of Health and Rehabilitation Sciences (project number: #2016SHRSOCT003), in accordance with the ethical review guidelines and processes of The University of Queensland. People were eligible to participate in the study if they: (a) were a parent of a child aged between four and twelve years, where the child had no diagnosed disabilities; (b) were aged 18 years or older; (c) lived in Australia; and (d) had sufficient English proficiency to understand and complete the questionnaire. Where parents were multiparous, they were asked to provide responses in relation to their youngest child.

Participants were invited to complete a self-administered questionnaire through a commercially available Internet survey instrument (SurveyMonkey), with the hyperlink distributed initially via the investigators’ personal social media forums (e.g., email, Facebook, Twitter) and other social groups (e.g., day care, school, gymnasium, church). Convenience sampling followed by a snowball approach was used, whereby participants were encouraged to inform other friends and groups about the study by word of mouth and social media. Information about the study was provided prior to obtaining consent and commencing the questionnaire. Completion of the questionnaire was voluntary, no incentive was provided, and anonymity was assured.

A total of 167 participants initially provided consent to participate in this cross-sectional study and were directed to the eligibility criteria. Data were excluded if the participants did not meet the eligibility criteria, or did not complete at least the parenting style section, and either the parental sensory sensitivity or adult attachment style sections, resulting in a study sample of 155 participants.

### Measures

#### Socio-demographic details

Socio-demographic details (age, gender, relationship status, number of children, ethnicity, employment status, carer responsibility, education and household income) were gathered.

#### Parenting styles: Parenting Styles & Dimensions Questionnaire

Parenting styles were measured using the Parenting Styles and Dimensions Questionnaire (PSDQ; [42]) used for children between 4–12 years. The PSDQ consists of 32 items rated on a 5-point Likert scale (0 = ‘never’ to 4 = ‘always’) to measure the three parenting styles: authoritative, authoritarian and permissive. Higher mean dimension scores reflect higher levels of each parenting style. An item indicative of *authoritative parenting* (high warmth and an appropriate level of control) would be, “I give my child reasons why rules should be obeyed”. An item indicative of *authoritarian parenting* (low warmth and high control) would be, “I scold and criticize to make my child improve”. Finally, an item indicative of *permissive parenting* (high warmth, low control) would be, “I find it difficult to discipline my child”. The validity and reliability of the PSDQ have been established in numerous studies [43]. Cronbach’s alphas in the present study were 0.89 for authoritative, 0.82 for authoritarian, and 0.69 for permissive parenting.

#### Sensory sensitivity: Highly Sensitive Person Scale-Shortened Version

The Highly Sensitive Person Scale-Shortened Version (HSPS-SV) is a self-report measure developed to measure sensory sensitivity in adults [44]. The HSPS-SV consists of 11 items derived from the original 27 items of the Highly Sensitive Person Scale [9], that are rated on an 8-point Likert scale (0 = ‘not at all’ to 7 = ‘extremely’). Items are summed to obtain an overall score, with higher scores indicating greater sensory sensitivity and lower scores indicating less sensory sensitivity (i.e., higher neurological threshold). Sensory sensitivity is characterized by sensitivity to both internal and external stimuli, including social and emotional cues. Sample items include, “Are you bothered by intense stimuli, like loud noises or chaotic scenes?”, and “Do you find yourself needing to withdraw during busy days into bed or into a darkened room or any place where you can have some privacy and relief from stimulation?”. Validity and reliability of the HPSP-SV was established in a recent study [7]. In the current study, the Cronbach’s alpha was 0.93.

#### Adult attachment style: Experiences in Close Relationships Scale-Modified 16

The Experiences in Close Relationship Scale-Modified 16-item scale (ECR-M16) is a previously validated self-report measure assessing adult attachment patterns, based on the original 36-item ECR [28]. While the original ECR asked questions in reference to romantic partners, the ECR-M16 asks individuals to report on thoughts, feelings and experiences in relation to people more generally to whom one feels close (e.g., friends, family) [45]. The items are rated on a 7-point Likert scale (1 = ‘disagree’ to 7 = ‘agree’), and summed to form two subscales: attachment anxiety and attachment avoidance. Higher scores indicate greater attachment insecurity. An item indicative of *attachment anxiety* would be, “I worry that other people won’t care about me as much I care about them”. An item indicative of *attachment avoidance* would be, “I get uncomfortable when other people want to be very close to me”. The ECR-M16 has shown adequate psychometric properties, including adequate reliability and validity [45]. In the current study, Cronbach’s alpha was 0.87 for attachment anxiety and 0.83 for attachment avoidance.

### Statistical analysis

Statistical analyses were performed using Version 24 of the Statistical Package for Social Sciences (SPSS), with a significance level set at 0.05 for this exploratory study. Descriptive statistics, including frequency distributions, were conducted and used for the purposes of data checking, and internal consistencies were calculated using Cronbach’s alpha. Pearson correlational analyses were computed between all continuous variables and a dichotomous variable, “employed or not”, to investigate associations among variables. Multiple regression analyses were calculated to consider the significance of relationships based on these results and to address the mediation hypotheses [46]. Mediation analysis using multiple regression was conducted to examine the capacity for parental sensory sensitivity to mediate associations between attachment and parenting style. The residuals of all regression models were checked for normality and homoscedasticity, and all assumptions were met.

To determine if parental sensory sensitivity mediated associations between attachment insecurity and parenting style, the four step-model proposed by Baron and Kenny [46] was utilized. First, the independent variable must be significantly related to the dependent variable (path c), the total effect; second, the independent variable must be significantly related to the mediator (path a); third, the mediator must be a significant predictor of the dependent variable, with the independent variable included in the analyses (path b); and finally, the previously significant relationship between the independent variable and dependent variable should be reduced or no longer significant (path c’), the direct effect. The indirect effect (path a * path b) is considered statistically significant, and mediation to have occurred, if the bootstrapped CI does not contain zero [47]. The PROCESS tool [48] in SPSS Version 24 was used to test the significance of the mediation effect, through 95% bootstrapped confidence intervals (CI), using 5000 samples.

## Results

### Demographic characteristics and subscale responses

As shown in Table 1, the majority of participants were White women who were tertiary educated and in a relationship. The participants were likely to be the primary carer or share equal responsibility for their youngest child, and almost half were working either part-time or full-time, with over 65% of household incomes over $100,000. Additional descriptive data and study variables are provided in Table 2.

**Table 1:** Descriptive details for demographic variables of the study sample (N = 155).

Variable		N	%
Parental/Carer gender		155	100.00
	Female	140	90.30
	Male	15	9.70
Relationship status		155	100.00
	In a relationship	138	89.00
	Not in a relationship	17	11.00
Number of children		155	100.00
	One child	24	15.50
	More than one child	131	84.50
Ethnicity		155	100.00
	White	143	92.30
	Not White	12	7.70
Employment status		155	100.00
	Employed	141	91.00
	Not employed	14	9.00
Level of carer responsibility		155	100.00
	Primary carer	87	56.10
	Spouse/partner is carer	7	4.50
	Equal responsibility	60	38.70
	Other	1	00.60
Education		155	100.00
	Up to and including year 12	12	7.70
	College/TAFE diploma	22	14.20
	Bachelor/honours/graduate diploma	86	55.50
	Masters or doctoral degree	35	22.60
Household income		155	100.00
	Up to $60,000	14	9.00
	$60,100 to $100,000	25	16.10
	$100,100 to $160,000	53	34.20
	$160,100 or more	49	31.60
	Undisclosed	14	9.00

**Table 2:** Descriptive data for continuous study variables.

Variable	N	Mean	Standard Deviation	Range
Parental/Carer age	155	41.41	5.17	28–55
Youngest child’s age	155	7.28	2.42	4–12
Parenting Styles				
PSQ authoritative	155	3.03	0.49	1.53–4.00
PSQ authoritarian	155	0.70	0.39	0.00–2.58
PSQ permissive	155	1.08	0.55	0.00–2.80
Adult attachment styles				
ECR attachment anxiety	149	16.50	9.26	0.00–47.00
ECR attachment avoidance	149	16.05	7.80	3.00–37.00
HSPS-SV	155	23.50	12.01	2.00–65.00

PSQ = Parenting Styles Questionnaire; ECR = Experiences in Close Relationships-Modified 16-item Scale; HSPS-SV = Highly Sensitive Persons Scale-Shortened version

### Correlations among variables

Results of Pearson’s correlation analyses between parenting styles (PSQ), sensory sensitivity (HSP-SV), and adult attachment (ECR-M16) are provided in Table 3. Higher levels of attachment anxiety and attachment avoidance were correlated with increased sensory sensitivity. Authoritarian and permissive parenting styles were positively correlated with sensory sensitivity and attachment anxiety. However, only permissive parenting was positively correlated with attachment avoidance. Authoritative parenting, in contrast, was not significant correlated with any of the attachment or sensory variables. No significant correlations were found between any demographic variables (e.g., age, gender, relationship status, number of children, ethnicity, employment status, carer responsibility, education and household income) and the outcomes variables (parenting). As a result, multivariate regression analyses examining the associations between parenting styles, sensory sensitivity and adult attachment, while controlling for demographic variables, were not conducted.

**Table 3:** Pearson correlations (r) between study variables (N = 155).

	PSQ authoritarian	PSQ authoritative	PSQ permissive	HSP-SV
HPS-SV	0.35***	-0.05	0.43***	-
ECR attachment anxiety	0.40***	-0.07	0.38***	0.50***
ECR attachment avoidance	0.09	-0.09	0.22**	0.29***

* = p< = 0.05;

**p< = 0.01;

***p< = 0.001

PSQ = Parenting Styles Questionnaire; ECR = Experiences in Close Relationships-Modified 16-item Scale; HSPS-SV = Highly Sensitive Persons Scale-Shortened Version.

### Mediation analyses with sensory sensitivity

Based on results of preliminary testing, three sets of variables were tested for mediation using the four step-model proposed by Baron and Kenny [46], as detailed below.

#### Attachment anxiety and authoritarian parenting style

**Fig 1:**
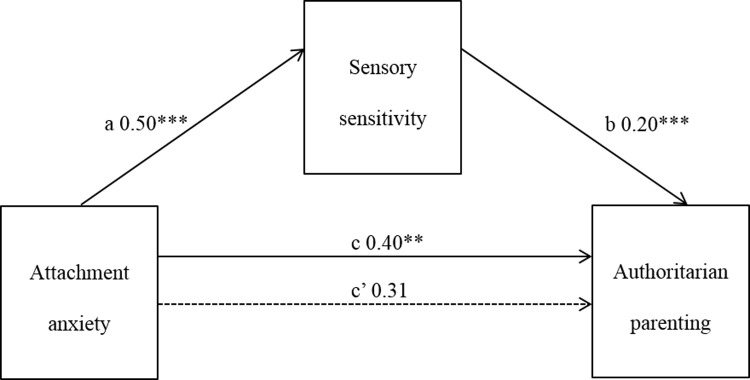
Mediation of attachment anxiety and authoritarian parenting by sensory sensitivity. * p< = 0.05; ** p< = 0.01; *** p< = 0.001.

The associations between attachment anxiety and authoritarian parenting, and attachment anxiety and parental sensory sensitivity, were both significant. When both the mediator (sensory sensitivity) and independent variable (attachment anxiety) were entered into a regression equation predicting authoritarian parenting, a significant result was obtained (adjusted *R*^2^ = .21, F[2,146] = 17.36, p < .001). Attachment anxiety accounted for most of this association, and while the direct effect of attachment anxiety on authoritarian parenting was reduced, it remained significant. Bootstrap estimation revealed the standardized indirect effect was significant (a*b = 0.10, CI = .0001, 0.0094) with sensory sensitivity partially mediating the relationship between attachment anxiety and authoritarian parenting style (Fig 1).

#### Attachment anxiety and permissive parenting style

**Fig 2:**
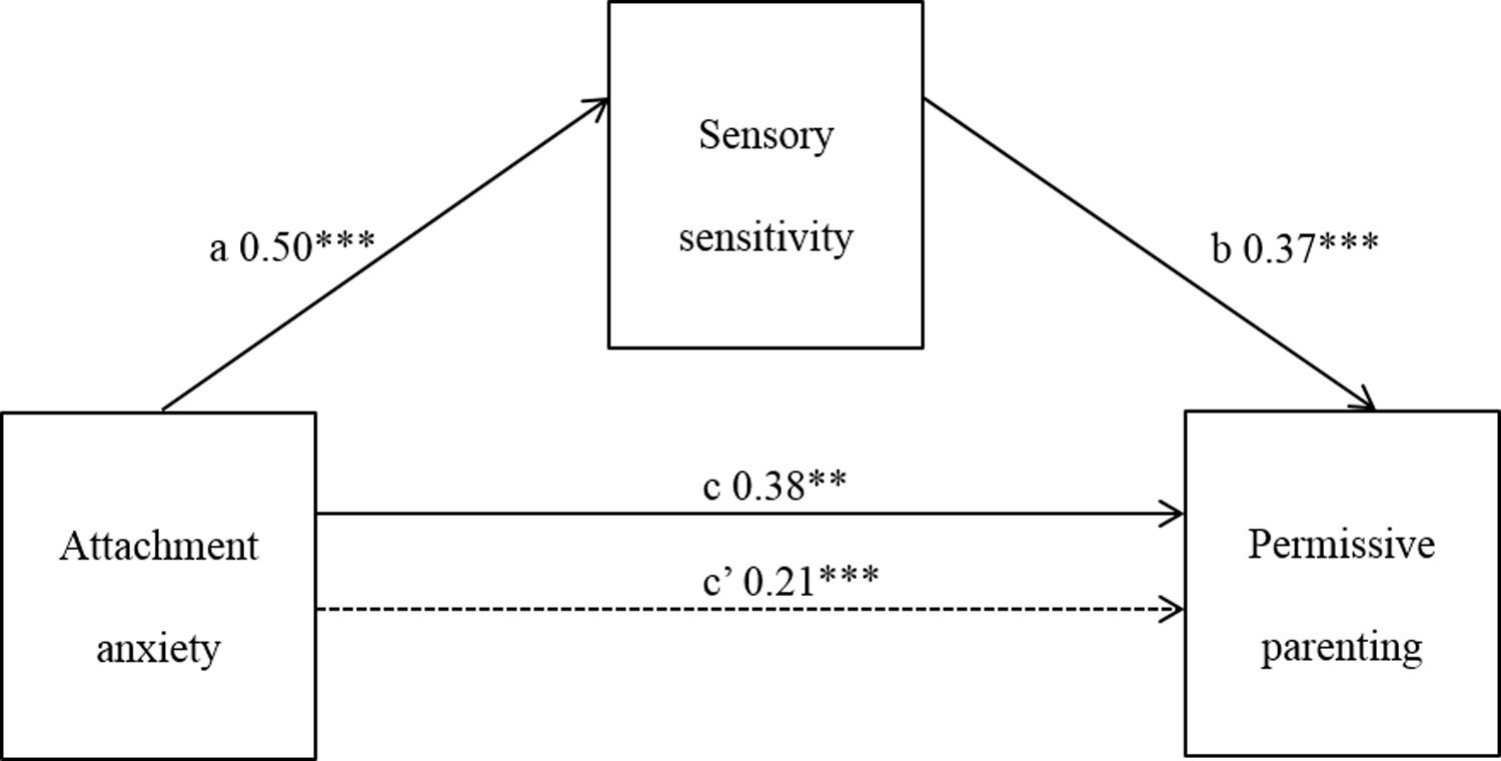
Mediation of attachment anxiety and permissive parenting by sensory sensitivity. * p< = 0.05; ** p< = 0.01; *** p< = 0.001.

The association between attachment anxiety and permissive parenting, and attachment anxiety and sensory sensitivity, were both significant. When both the mediator (sensory sensitivity) and independent variable (attachment anxiety) were entered into a regression equation predicting permissive parenting, a significant result was obtained (adjusted R^2^ = .21, F[2,146] = 20.50, p < .001). Sensory sensitivity accounted for most of this association, and the direct effect of attachment anxiety on permissive parenting was reduced but remained significant. Bootstrap estimation revealed the standardized indirect effect was significant (a*b = 0.19, CI = .0001, .0030) with sensory sensitivity partially mediating the relationship between attachment anxiety and permissive parenting style (Fig 2).

#### Attachment avoidance and permissive parenting style

**Fig 3:**
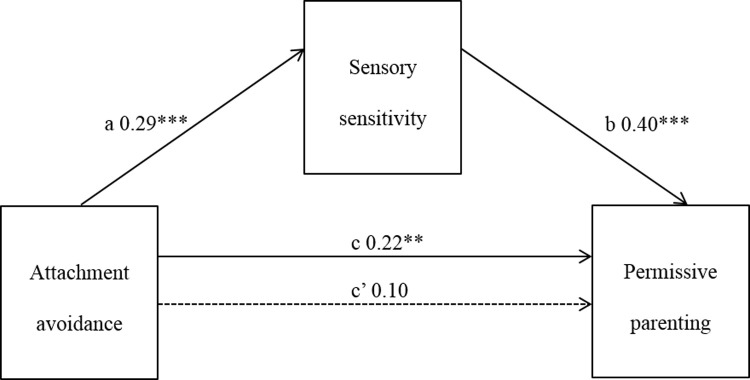
Mediation of attachment avoidance and permissive parenting by sensory sensitivity. * p< = 0.05; ** p< = 0.01; *** p< = 0.001.

The association between attachment avoidance and permissive parenting, and attachment avoidance and sensory sensitivity, were both significant. When both the mediator (sensory sensitivity) and independent variable (attachment avoidance) were entered into a regression equation predicting permissive parenting, a significant result was obtained (adjusted R^2^ = .18, F[2,146] = 17.63, p < .001). Sensory sensitivity accounted for most of this association, and the direct effect of attachment avoidance on permissive parenting was reduced and no longer significant. Bootstrap estimation revealed the standardized indirect effect was significant (a*b = 0.12, CI = .0018, .0112), with sensory sensitivity fully mediating the relationship between attachment avoidance and permissive parenting style (Fig 3).

## Discussion

The aim of this study was to examine the relationships between sensory sensitivity, adult attachment patterns with significant others (e.g., friends, family, partners), and Baurmind’s parenting styles. To our knowledge, this is the first quantitative study to consider parental sensory sensitivity when investigating parenting styles, and the first to evaluate the potential mediating role of sensory sensitivity in the relationship between attachment insecurity and parenting styles. Overall, results were largely supportive of hypotheses, and are discussed in detail below.

### Adult attachment and parental sensory sensitivity

Supporting Hypothesis 1, higher levels of both insecure attachment patterns were related to greater sensory sensitivity. This finding implies that insecurely attached parents are more likely to become overwhelmed with sensory stimuli, such as loud or unexpected noise, interpersonal contact, mess, and tantrums. These findings are consistent with previous evidence that anxious attachment and sensory sensitivity are linked in adult populations [5–8]. Results relating to attachment avoidance and sensory sensitivity continue to be mixed. Evidence from the current study that they were linked is consistent with the study by Meredith et al. [7], but contrasts with other studies [5, 6, 8] described earlier, which did not find links between attachment avoidance and sensory sensitivity. Interestingly, Meredith et al. [7] found that associations between attachment anxiety and sensory sensitivity were not retained when controlling for distress variables (such as anxiety, depression and stress), whereas associations between attachment avoidance and sensory sensitivity were unrelated to any distress variables. Meredith et al. [7] therefore suggested that, for individuals who respond passively to sensory information, education regarding how to actively regulate sensory information may help minimize distress for a person who is insecurely attached. Such education and coaching to assist individuals to modify their coping strategies may be particularly beneficial as passive coping strategies, similar to pain [38] and mental health literature [49], are considered less adaptive than active coping strategies given that sensory information is unimpeded and continues to irritate and disrupt the functioning of an individual. In contrast, the behaviors associated with authoritarian and permissive parenting styles may represent coping strategies for sensitive people (who may be insecurely attached). This proposition is discussed below.

### Adult attachment and parenting style

Consistent with Hypothesis 2, parents who reported higher levels of insecure attachment also reported higher levels of authoritarian and permissive parenting styles. More specifically, attachment anxiety was associated with both authoritarian and permissive parenting styles, whereas attachment avoidance was associated only with permissive parenting. These findings are largely consistent with findings of two other studies in which parental attachment pattern and Baumrind’s typology were considered [32, 34]. Similarly, in an extensive review of research involving over 60 studies, Jones, Cassidy and Shaver [4] concluded that associations exist between insecure attachment patterns and negative parenting behaviors (e.g., less warmth, supportiveness and responsiveness).

When examining parenting literature more broadly to support understanding of these associations, anxious attachment has been associated with hyper-vigilance and increased reactivity in parenting confrontations regarding a child’s undesirable behavior [26, 50], which may be reflective of the higher control dimension of the authoritarian parenting style. Attachment anxiety has also been linked with a strong desire for closeness and intimacy [26, 50], which may be reflective of the higher warmth dimension of the permissive parenting style. Additionally, given that avoidantly attached individuals tend to detach themselves emotionally (and physically) to reduce their discomfort of expressions of need or dependence [26, 50], such parents may be less controlling or distance themselves from their child, which may be akin to the lower control dimension of the permissive parenting style.

### Parental sensory sensitivity and parenting style

Parents who reported higher levels of sensory sensitivity also reported more authoritarian or permissive parenting styles. This finding is consistent with Hypothesis 3, and offers new insights into possible factors influencing parenting style. A person who is more sensory sensitive has lower thresholds for sensory stimuli; thus, they may more easily notice even small amounts of sensory information, and become more easily overwhelmed. Accordingly, a strategy for a parent who is more sensory sensitive may be to enforce strict routines and discipline to control the level and predictability of sensory input (i.e., authoritarian parenting). Alternatively, parents may ignore or move away from the sensory input and allow the child to behave as they please to avoid sensory overload (i.e., permissive parenting). Our findings are consistent with the qualitative findings of Turner [15] who identified examples of coping strategies used by mothers with sensory processing challenges.

### Parental sensory sensitivity, adult attachment and parenting style

As predicted in Hypothesis 4, sensory sensitivity was not only associated with authoritarian and permissive parenting styles, it also mediated the relationship between attachment insecurity and both authoritarian and permissive parenting styles. When examining attachment *anxiety*, sensory sensitivity was found to partially mediate the relationship between attachment anxiety and both authoritarian and permissive parenting. That is, parents who are anxiously attached are more likely to respond to their children with less warmth and more (authoritarian parenting style) or less (permissive parenting style) control, and particularly when sensory sensitive. When exploring attachment *avoidance*, sensory sensitivity was found to fully mediate the relationship between attachment avoidance and permissive parenting. This finding suggests that avoidantly attached parents may display more authoritarian or permissive parenting styles due to their level of sensory sensitivity. A possible reason for this finding is that an individual’s reactivity to sensory stimuli may influence their reaction towards complex environmental events and stressors, such as parenting. Parenting children may be particularly sensorally stimulating, and possibly overwhelming, given that children may be intense in their affective responses (e.g., auditory stimuli from crying, proprioceptive feedback from hitting their parents, visual stimuli from throwing objects), particularly during stressful events. Children also engage in multi-sensory activities (e.g., finger-painting in the house which primarily provides tactile and visual stimuli, playing in the playground which primarily provides vestibular and auditory stimuli) that parents are required to manage and participate in. Overall, these findings suggest that sensory sensitivity may possibly mediate the relationship between adult attachment and parenting styles, and that parents may adopt particular parenting styles as a way to cope with their relationship and sensory needs.

### Limitations and considerations

There are a number of limitations of this study that should be considered. All measures were self-report and, although completed anonymously, may have been open to bias from factors such as memory, mood, disposition, and social desirability. While no objective measures were used that may have corroborated the self-report data, the strength of using self-report measures is that it assists in providing insights into how the participants experience sensory information, perceive their own parenting, and view the trustworthiness of others. Furthermore, convenience sampling may have contributed to selection bias, with most of the sample being White mothers who were well-educated and in relationships. A replication of the study with a random sample of diverse parents and children (e.g., culturally and linguistically diverse backgrounds, poorer socio-economic backgrounds, more fathers, single parents) is recommended to improve the generalizability of results. While the majority of the sample were also women (i.e., mothers), results did not change when men (i.e., fathers) were excluded from analyses. Results may differ with statistical consideration of additional confounding variables related to demographic characteristics (e.g., prematurity of child, child gender), parental mental health, and the child’s sensory processing pattern through an observational study of parent-child dyads. The method of measuring adult attachment in this study is well accepted; however, it taps only attachment avoidance and anxiety. Using an attachment measure that specifically measures attachment security, such as the Attachment Styles Questionnaire [51], will support comparisons with literature in the attachment/health field. In addition, inclusion of the Adolescent/Adult Sensory Profile [16], or objective sensory testing, in future research will provide a richer insight into the influence of parental sensory sensitivity on parenting styles. Finally, given these preliminary findings, the complex interrelationships between sensory processing patterns, adult attachment, and parenting styles warrant future longitudinal investigations to inform clinical approaches.

### Clinical implications

Study findings provide preliminary support for the value of considering sensory sensitivity along with attachment during parenting-related assessment, support and treatment to facilitate the development of favorable parenting styles. Currently, many parenting programs focus on the parent’s attachment system but not the parent’s sensory sensitivity to support the development of adaptive parenting styles and ultimately mitigate child behavioral difficulties [52, 53]. While it is plausible that sensory modulation strategies may be helpful to improve parenting styles, or that a parent’s sensory sensitivity may influence the capacity of parenting styles to change, further research is needed to elucidate these claims.

More specifically, results of the present study may assist in the early identification of parents at risk of suboptimal parenting styles (e.g., authoritarian and permissive styles) by assessing for parental sensory sensitivity. While preliminary evidence suggests that interventions such as “coaching” [54], sensory activity scheduling [55], and “Mediational Intervention for Sensitizing Caregivers” [56] may assist parents to understand and manage their *children’s* sensory patterns, further research is needed to understand if similar approaches may also assist parents to understand and manage their own sensory sensitivity leading to more adaptive parenting styles. By supporting parents to develop more favorable parenting styles, this may have positive implications on family well-being and developmental outcomes for the child both in the short- and longer-term [43].

The findings also provide a possible explanation for some of the inconsistent reports in the literature regarding measures of attachment and parenting behaviors [4]. For example, while some studies have shown that parents with insecure attachment patterns do not report less favorable parenting behaviors, others have revealed significantly less favorable parenting behaviors among insecurely attached parents [4]–perhaps sensory sensitivity is the necessary explanatory variable.

## Conclusions

Although there have been earlier theoretical discussions [13] and one qualitative study [15], to the authors’ knowledge, this study is the first to provide quantitative evidence of the associations between parental sensory sensitivity and parenting styles. In addition, while evidence relating adult attachment styles to parenting styles is well established, our research provides preliminary evidence that parental sensory sensitivity may influence this association. Parental sensory sensitivity was associated with attachment insecurity and with more authoritarian and permissive parenting styles, and also mediated the associations between attachment insecurity and both the authoritarian and permissive parenting styles. Interventions that increase a parent’s capacity to modulate and cope with sensory stimuli may temper the influence of insecure attachment on parenting. Further research is indicated to provide support of our findings and to examine implications for intervention and prevention.

## Supporting information

S1 File Supporting data file. Data of participants in the research study.
(SAV)
